# Editorial: Advances in the Understanding of Tumor Microenvironment: Molecular and Theranostic Imaging

**DOI:** 10.3389/fbioe.2021.731119

**Published:** 2021-08-05

**Authors:** Shouju Wang, Hui Mao, Zhen Cheng, Zhaogang Teng, Xiaolian Sun

**Affiliations:** ^1^ Department of Radiology, The First Affiliated Hospital of Nanjing Medical University, Nanjing, China; ^2^ Department of Radiology and Imaging Sciences, Emory University School of Medicine, Atlanta, GA, United States; ^3^ Molecular Imaging Program at Stanford, Department of Radiology and Bio-X Program, Canary Center at Stanford for Cancer Early Detection, Stanford University, Stanford, CA, United States; ^4^ Key Laboratory for Organic Electronics and Information Displays, Jiangsu Key Laboratory for Biosensors, Institute of Advanced Materials, Jiangsu National Synergetic Innovation Centre for Advanced Materials, Nanjing University of Posts and Telecommunications, Nanjing, China; ^5^ State Key Laboratory of Natural Medicines, Key Laboratory of Drug Quality Control and Pharmacovigilance, Department of Pharmaceutical Analysis, China Pharmaceutical University, Nanjing, China

**Keywords:** tumour microenvironment, molecular imaging, theranostics, immune microenvironment, nanoprobes

Tumor microenvironment (TME) plays key roles in cancer development, prognosis and responses to treatments. However, given its heterogeneity and complex interactions/interplays with other malignant processes, investigating TME requires systematical and preferably non-disruptive approaches. Molecular imaging enables visualizing, characterizing, and even quantitatively measuring specific biological and molecular processes *in vivo* to monitor the heterogeneity and evolution of TME. Theranostic imaging, as a rapidly expanding facet of molecular imaging, also creates new possibilities to deploy treatment options targeting TME by delivering therapeutic agents or disruptive photothermal and photodynamic energy. The advanced molecular and theranostic imaging tools and approaches presented in this special issue, including one review article, one mini review article, and two original research articles, offer an overview and a few examples of such applications.

Focusing on immune therapy that aims to reverse the immune-suppressive TME, Li et al. provided a latest overview of the recent progress of breast cancer treatments, including the predictive biomarkers, immune checkpoint blockades, cancer vaccines, and adoptive cell therapy, which directly or indirectly changed TME to exert the efficacy. In this mini review article, the authors highlighted the potential of nanotechnology to monitor and control the tumor immune microenvironment. Gao et al. introduced the therapeutic applications of gold nanoparticles in cancer treatment and discussed the potential of using stimuli-responsive strategies to respond to TME and to increase the transport efficacy. Serval unique properties, such as localized surface plasma resonance and stimuli-responsive properties to hypoxia and acidic pH, making gold nanoparticles promising for transport therapeutic drugs to TME. In a research report, Heaster et al. demonstrated intravital metabolic autofluorescence imaging can be used to resolve the dynamic macrophage function and reveal the heterogeneity of macrophage between normal and cancerous microenvironments *in vivo*. This method enabled autofluorescence imaging to monitor macrophage behavior in response to treatment, which would be helpful to identify new targets for drug development. Finally, Wang et al. present a rationally designed sandwich nanostructure of gold-graphene hybrid material that can be used for photoacoustic image-guided photothermal therapy. The imaging-guided photothermal therapy could precisely control the temperature of the tumor and potentially alter the TME through the disruption of its specific components.

As this Research Topic on this issue covers the recent advances in molecular and theranostic imaging of TME, it should be noted that the potential of nanotechnology and molecular imaging applications in imaging and modulation of TME has yet to be fully explored. The editors hope that the selected articles on this Research Topic will inspire future work to further advance and expand molecular and theranostic imaging in studying TME and beyond.

